# Nutrition in Pregnancy Following Bariatric Surgery

**DOI:** 10.3390/nu9121338

**Published:** 2017-12-08

**Authors:** Christopher Slater, Lauren Morris, Jodi Ellison, Akheel A. Syed

**Affiliations:** 1 Department of Nutrition and Dietetics, Salford Royal NHS Foundation & University Teaching Trust, Salford, Greater Manchester M6 8HD, UK; chris.slater@srft.nhs.uk; 2 Department of Diabetes & Endocrinology, Salford Royal NHS Foundation & University Teaching Trust, Salford, Greater Manchester M6 8HD, UK; Lauren.morris@srft.nhs.uk; 3 Department of Surgery, Salford Royal NHS Foundation & University Teaching Trust, Salford, Greater Manchester M6 8HD, UK; jodi.ellison2@srft.nhs.uk; 4 Faculty of Biology, Medicine & Health, The University of Manchester, Manchester M13 9PL, UK

**Keywords:** bariatric surgery, pregnancy, nutritional status

## Abstract

The widespread use of bariatric surgery for the treatment of morbid obesity has led to a dramatic increase in the numbers of women who become pregnant post-surgery. This can present new challenges, including a higher risk of protein and calorie malnutrition and micronutrient deficiencies in pregnancy due to increased maternal and fetal demand. We undertook a focused, narrative review of the literature and present pragmatic recommendations. It is advisable to delay pregnancy for at least 12 months following bariatric surgery. Comprehensive pre-conception and antenatal care is essential to achieving the best outcomes. Nutrition in pregnancy following bariatric surgery requires specialist monitoring and management. A multidisciplinary approach to care is desirable with close monitoring for deficiencies at each trimester.

## 1. Introduction

Excess weight is a major public health problem in Western societies, with overweight defined as a body mass index (BMI) ≥ 25 kg/m^2^, and clinical obesity defined as a BMI ≥ 30 kg/m^2^, affecting men and women across all age groups. Approximately one half of women of childbearing age in England are overweight or obese, with a similarly high prevalence in several European countries, the USA and Australia [1,2,3,4]. Obesity is associated with an increased risk of developing various medical conditions including type 2 diabetes mellitus (T2DM), coronary artery disease, fatty liver disease, obstructive sleep apnea and osteoarthritis [5]. In addition to these health risks, women who are obese are more likely to encounter issues with subfertility or infertility [6,7,8,9]. They are also more likely to suffer complications during pregnancy including stillbirth, gestational diabetes, pre-eclampsia, thromboembolism and maternal death [10,11]. Obese pregnant women are also more likely to have caesarean sections, post-partum hemorrhage and poor wound healing [12]. While dietary and lifestyle modifications remain first line in the management of obesity, a more widespread use of bariatric surgery is being observed across the world [13,14,15]. Bariatric surgery has been shown to achieve sustained weight loss [16,17], and improvements in several obesity-related conditions including T2DM, hyperlipidemia, hypertension and obstructive sleep apnea [18]. It has also been shown to be a cost-effective treatment for obesity [19,20]. Globally, more women than men undergo bariatric surgery [14,18,21], commonly in women of childbearing age [13,22,23]. Whilst bariatric surgery can improve fertility and maternal outcomes for obese women [24,25,26], patients may encounter complications during pregnancy attributable to bariatric surgery [27]. In this narrative review, we discuss nutritional aspects of post-bariatric surgery care during pregnancy.

### Bariatric Surgery

**Figure 1:**
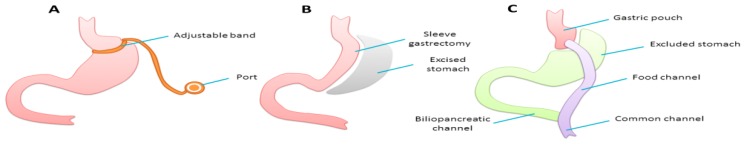
Common types of bariatric surgery: adjustable gastric banding (**A**); sleeve gastrectomy (**B**); and Roux-en-Y gastric bypass (**C**).

Weight loss operations are commonly classified as restrictive, malabsorptive or varying combinations of the two. Laparoscopic adjustable gastric banding, a restrictive operation, involves the placement of an inflatable silicone band wrapped around the top part of the stomach to create a small pouch above and a narrow opening to the main body of the stomach below (Figure 1A); the band can be inflated by injection of sterile water through a subcutaneous port connected to the band by tubing. Sleeve gastrectomy is also a restrictive procedure that involves surgical removal of the greater curvature of the stomach to create a narrow gastric sleeve (Figure 1B). In Roux-en-Y gastric bypass, a small pouch is created at the gastric fundus, restricting food intake; the main body of the stomach and a length of duodenum and jejunum are bypassed from the food channel, thereby creating an element of malabsorption as well (Figure 1C). Single anastomosis gastric bypass is a recent innovation that reduces the technical complexity of the traditional gastric bypass operation. Other types of bariatric surgery such as biliopancreatic diversion (BPD) and duodenal switch (DS) cause significant malabsorption and are less commonly performed.

## 2. Materials and Methods

We undertook a focused, non-systematic, narrative review with searches of the published literature in PubMed (www.pubmed.gov) and Google Scholar (www.scholar.google.com) with a broad range of combinations of the medical subject headings (MeSH) terms, ‘obesity’, ‘maternal obesity’, ‘bariatric surgery’, ‘gastric bypass’, ‘gastric band’, ‘sleeve gastrectomy’, ‘pregnancy’ and ‘fertility’; English language articles retrieved up to November 2017 were included. Where high-quality published literature was unavailable we have made pragmatic recommendations based on our own expertise and experience in the field.

## 3. Results

The majority of women undergoing bariatric surgery are of reproductive age and many start or complete their families following bariatric surgery. Optimal care for these women should include pre-conception counselling, optimization of nutrition before, during and after pregnancy, and monitoring for complications.

### 3.1. Pre-Conception Care

Whereas obesity in women of childbearing age is linked with lower fertility rates, overall fertility improves after bariatric surgery [28]. As the first year after bariatric surgery is characterised by an active catabolic state, with gradual stabilization of nutritional status in the following months, women are generally advised to avoid pregnancy for 12–24 months after bariatric surgery [27]. This aims to reduce the risk of intrauterine growth retardation whilst allowing the woman to achieve the full weight loss and metabolic outcomes of the bariatric surgery. Pre-conception counselling should include consideration of non-oral contraceptives during this time as absorption of oral contraceptive pills may be compromised following bariatric surgery [29].

Several international societies have advised on the standards of usual post-operative care for all persons who have undergone bariatric surgery [30,31,32,33,34]. Recommended nutrient supplements commonly include multivitamins and micronutrients, calcium and vitamin D, iron and vitamin B12 (Table 1). However, not all supplements may be suitable during pregnancy and lactation. For instance, retinol-based vitamin A products are best avoided in pregnancy due to their teratogenic potential. Pre-conception assessment should therefore include specialist dietetic review, including appropriate multivitamin and micronutrient supplements and adequate dietary protein intake. Additional folic acid supplementation is also routinely advisable (see Section 3.2.5).

### 3.2. Antenatal Care

Common nutrient deficiencies following bariatric surgery can be exacerbated by pregnancy symptoms such as morning sickness or hyperemesis, gastro-esophageal reflux, abdominal bloating and pressure symptoms. Compliance may also be a concern, with studies reporting poor adherence with nutritional supplementation post-operatively [35,36]. Women who become pregnant following bariatric surgery should have nutritional surveillance and laboratory screening for deficiencies every trimester [31,32].

#### 3.2.1. Dietary Assessment

Following bariatric surgery, patients are advised to adopt a regular eating pattern, aiming to include three small, balanced meals per day that include protein, vegetables/salad and a small amount of carbohydrate. Snacks can be included between meals if patients feel hungry—these should be appropriate in size and texture and help to achieve a balanced intake. These patterns should be encouraged after pregnancy to ensure that sufficient nutrients are consumed to meet the requirements of mother and baby.

Nausea and vomiting are common in pregnancy, affecting up to 80% of pregnant women [37]. Early management with antiemetic’s and dietary advice is recommended to avoid or treat complications [37]. The advice to eat little and often is of greater importance in these patients; increased intake of nutritious fluids to help meet requirements may be necessary. Ginger could be used as a complementary therapy [37], but may not be effective for all patients. Other dietary and behaviour changes to improve nutritional intake when nauseous can include:

- Taking anti-emetics as prescribed, not waiting for the onset of nausea
- Avoiding foods that induce nausea
- Avoiding cooking smells and other strong odours
- Taking a short walk before meals
- Tracking nausea to establish any patterns and maximising nutritional intake when typically less nauseous
- Experimenting with different foods as taste changes can occur over time
- Following good eating habits by
  - Separating consumption of solids and liquids
  - Taking small bites
  - Chewing well
  - Stopping eating when full

#### 3.2.2. Energy Requirements

Recommendations for energy intake during pregnancy increase by 200 kcal/day, but only in the final three months of pregnancy [38]. The balance of energy is thought to be met by reduced activity, although an increased weight will mean more energy being used by completing the same tasks [39]. Managing expectations of weight before, during and after pregnancy is important. From our clinical experience, patients are often worried about weight gain during and after pregnancy. This can be a psychological barrier for some patients to eating a balanced diet with sufficient energy and protein. On the other hand, some patients may succumb to the misconception of ‘eating for two’ and suffer from excess weight regain. Pregnant women should avoid any further weight loss during pregnancy, instead focusing on a balanced, varied dietary intake. Weight gain during pregnancy of 6 kg in those with a BMI > 25 kg/m^2^ should be expected, and no more than 12 kg in women of a healthy BMI [39].

#### 3.2.3. Protein Intake

Protein deficiency is uncommon but may occur due to reduced intake, intolerance of protein-rich foods or procedures that lead to malabsorption such as biliopancreatic diversion or a Roux-en-Y gastric bypass with longer roux limb length [40]. In addition to concerns regarding fetal growth, low protein intake may be associated with higher childhood blood pressure and risk of cardiovascular disease in later life [41,42]. Protein deficiency is also associated with poor wound healing, which may be of particular concern as caesarean section rates are higher amongst obese women. Clinically significant hypoalbuminemia may present with edema, which could be misinterpreted as a normal symptom of pregnancy or as a sign of pre-eclampsia by those unfamiliar with this potential consequence of bariatric surgery.

A reference nutrient intake of 0.75 g of protein per kg of body weight plus an additional 6 g/day in pregnancy is recommended [38]. This target can be difficult to achieve for patients who remain overweight or obese. Therefore, a minimum intake of 60 g of protein per day is universally recommended [31,43,44], but tailored to the individual patient. Food and fluid fortification techniques may help to increase the protein intake of the usual diet.

#### 3.2.4. Food Hygiene

It is important to reiterate pregnancy food safety advice to avoid complications such as gastroenteritis and toxicity. This includes avoiding:

- Soft, ripe and blue veined cheeses such as brie and stilton.
- Other non-pasteurized dairy products including soft ice creams.
- Shark, marlin and swordfish due to mercury content.
- Liver and liver products such as pate and cod liver oil to avoid consumption of too much retinol-based vitamin A.
- Eggs, unless certified salmonella-safe, should be cooked thoroughly until the yolk and white are solid [45]; unpasteurized foods containing uncertified raw or lightly cooked eggs, such as homemade mayonnaise, salad dressings, custard and eggnog, should be avoided.

#### 3.2.5. Micronutrients

Deficiencies of micronutrients are common following bariatric surgery and may be exacerbated during pregnancy due to pregnancy-related symptoms and increased maternal and fetal demand.

##### Iron

Iron deficiency is the most commonly encountered deficiency following bariatric surgery, occurring in nearly one half of patients, particularly after Roux-en-Y gastric bypass or biliopancreatic diversion surgeries [40]. Mechanisms for iron deficiency post-bariatric surgery include reduced gastric pH, limited intestinal absorption, avoidance of iron-rich foods, reduced gastric acid secretion, use of proton pump inhibitors, and the exclusion of the duodenum and jejunum (primary sites of absorption). Women of childbearing age are most at risk due to menstrual losses. Iron requirement increases to 800 milligrams of elemental iron of which around 300 milligrams is required by the fetus and placenta and the remaining 500 milligrams for maternal hemoglobin mass expansion [46]. There is evidence that maternal iron deficiency leads to an increased risk of pre-term delivery and subsequent low birth weight [47]. Caesarean sections and postpartum hemorrhages are more frequent amongst obese women [48], and iron deficiency anemia in these circumstances could lead to additional increased harm. Iron supplements may cause gastrointestinal side-effects including nausea and constipation, which may exacerbate pre-existing symptoms associated with pregnancy and/or bariatric surgery, thus making compliance with supplements difficult.

Current guidelines recommend that patients undergoing gastric bypass, sleeve gastrectomy and biliopancreatic diversion/duodenal switch operations should be supplemented with 45–60 mg elemental iron [30]. This should increase to 100 mg of elemental iron per day for menstruating women [32]. Additional supplementation with vitamin C to aid absorption should be considered in those with persistently low iron levels. Monitoring of iron status should be undertaken at regular intervals. Intravenous iron may be needed in patients where oral iron preparations have failed to correct deficiency [31].

##### Calcium and Vitamin D

Calcium homeostasis alters in pregnancy to meet the rising demands of calcium from the growing fetus to ensure adequate mineralization of the fetal skeleton, and in preparation for lactation. Increased levels of 1,25-dihydroxy vitamin D enhance absorption of calcium from the intestine to regulate maternal serum calcium. Other mechanisms to regulate maternal calcium in pregnancy include reduced urinary excretion of calcium and increased resorption of calcium from the maternal skeleton. The latter has raised concerns regarding increased risk of fracture following pregnancy, but there is insufficient evidence regarding whether significant bone loss occurs [49]. A recent review addresses the effects of bariatric surgery on bone health [50]. However, whether pregnancy following bariatric surgery has a synergistic effect on bone loss is unknown. During lactation, 300–400 milligrams of calcium are lost into breast milk per day [51]. Increased parathyroid hormone-related peptide levels and relatively low estrogen levels could cause levels of bone loss comparable with menopause, but changes are usually quickly reversed following weaning and are unlikely to have long-term consequences for otherwise well women [49,51].

The primary source of calcium in pregnancy is dietary, but antacids and supplements containing calcium carbonate, and prenatal vitamins, also contribute. Whilst the reference nutrient intake for calcium in pregnant and lactating women is 700 mg per day [38], a calcium intake of 1500 mg daily in women who have had bariatric surgery is recommended [44]. This may be particularly difficult to achieve without supplementation. Whilst some authors have advocated calcium citrate in preference to calcium carbonate due to potentially better absorption when reduced gastric acid is expected post-bariatric surgery [52], the latter remains the most widely prescribed formulation. Patients are advised not to take their iron and calcium supplements together to ensure adequate absorption.

Vitamin D is required to support calcium metabolism and deficiency is linked to rickets in infancy, fetal growth restriction and pre-eclampsia [49]. Whilst vitamin D requirements in pregnancy are not altered, with a reference nutrient intake of 20 micrograms (800 IU) daily [38], pre-existing deficiency is common. Vitamin D supplement doses vary according to the baseline vitamin D, but larger doses are likely to be required following gastric bypass, gastric sleeve or duodenal switch procedures, due to reduced absorption. After these procedures, routine supplementation with 20 micrograms (800 IU) of vitamin D per day is recommended [32].

##### Folic Acid

Folate (vitamin B9) has essential roles in DNA and RNA synthesis and single-carbon transfer reactions. Deficiency can cause megaloblastic anemia, thrombocytopenia, leucopenia, glossitis and elevated homocysteine levels. Adequate folate intake is essential to prevent neural tube defects in the fetus. Folic acid deficiency can occur following bariatric surgery due to low gastric pH and reduced capacity for intestinal absorption [53]. Folate can be absorbed in the remaining small intestine and widespread fortification of foods such as breakfast cereals means that folate deficiency following bariatric surgery is relatively uncommon [40]. However, fetal myelomeningocele has been reported in a post-gastric bypass patient who was non-compliant with supplementation [54].

Pre-conception supplementation with 400 micrograms per day of folic acid and continuing until the 12th week of pregnancy is recommended [55]. This increases to 5 mg in women at higher risk, including those with obesity and/or diabetes and those with a history of prior pregnancies complicated by neural tube defects. Women who have undergone bariatric surgery should be deemed as high-risk. As such we advise an additional 5 mg of folic acid daily preconception and for the first 12 weeks of the pregnancy.

##### Vitamin B12

Vitamin B12 (cobalamin) functions as an essential co-enzyme in several important enzyme-catalyzed reactions. Deficiency of vitamin B12 can lead to megaloblastic anemia and potentially irreversible neurological damage and fetal neural tube defects. Vitamin B12 deficiency is common following bariatric surgery due to reduced availability of intrinsic factor and malabsorption. Patients undergoing procedures which exclude the greater curvature of the stomach, such as gastric bypass, sleeve gastrectomy and biliopancreatic diversion/duodenal switch are at particular risk [31]. In one study, deficiency was reported in over 50% of pregnancies occurring post-bariatric surgery [56]. Following bariatric surgery, intramuscular injections of hydroxocobalamin 1 mg at three monthly intervals are recommended [32,57].

##### Vitamin A

The general pregnant population is usually advised to avoid vitamin A supplementation due to the risk of fetal malformation with retinol isoforms [58]. However, as a fat-soluble vitamin, vitamin A deficiency can be encountered due to malabsorption following bariatric surgery. Maternal vitamin A deficiency has been shown to cause night blindness and is associated with increased maternal mortality and pre-term birth [59]. It is therefore recommended that pregnant women and those planning pregnancy following bariatric surgery are supplemented with vitamin A in the beta carotene form [32].

##### Thiamine

Patients with decreased nutritional intake, increased nutrient losses or impaired nutrient absorption are at risk of thiamine deficiency [60]. Patients who have had bariatric surgery are therefore already at risk of deficiency, as are patients with hyperemesis [60]. Hyperemesis Gravidarum (HG) affects 0.3–3.6% of pregnant women [61]. It is characterised by severe nausea and vomiting associated with weight loss of 5% of pre-pregnancy weight, dehydration and electrolyte imbalance. The incidence of HG in post-bariatric surgery pregnant women is unknown. Anecdotal case studies have reported gastric band slippage after HG [62,63]. In our experience, this may require support with parenteral nutrition and eventual removal of the gastric band.

Wernicke’s encephalopathy is a well-known complication of thiamine deficiency. It has been reported in a woman with HG following gastric bypass surgery [64]. Additional thiamine supplementation (either oral or intravenous) is recommended in patients with prolonged vomiting, especially prior to administration of dextrose or parenteral nutrition [32,37]. It is advisable to start replacement therapy as soon as possible when a diagnosis of Wernicke’s encephalopathy is suspected to prevent progression to an irreversible condition, even before the diagnosis is confirmed [64].

#### 3.2.6. Dumping Syndromes

Dumping syndromes can occur following gastric bypass or sleeve gastrectomy surgeries. Early dumping, which occurs within one hour of eating, is attributed to rapid transit of ingested food to the small bowel resulting in release of vasoactive hormones [27]. Symptoms include abdominal pain, nausea, diarrhea, flushing, palpitations, tachycardia and hypotension. The mainstay of management is dietary measures, including advising patients to consume smaller amounts in one sitting by dividing the recommended daily food intake between six meals [65]. Patients are also advised to postpone intake of liquids for at least 30 min after a meal. Lying down for 30 min after meals can help to reduce vasomotor symptoms by slowing down gastric emptying.

Late dumping or post-prandial hypoglycemia occurs 1–3 hours after eating carbohydrates. It is partly incretin-mediated and is thought to occur in response to hyperinsulinemia following rapid glucose transit into the jejunum. This causes a reactive hypoglycemia characterized clinically by diaphoresis, tremors, impaired mental concentration, altered consciousness, palpitation and sometimes syncope [27]. Dietary changes are the mainstay of management and include a low glycemic index diet with avoidance of refined carbohydrates. The choice of pharmacotherapy in pregnant women is limited. Adding pectin or guar gum to increase the viscosity of food, which slows down gastric emptying, can ameliorate symptoms in the short term [65]. Our group has previously reported acarbose treatment in one woman with intractable post-prandial hypoglycemia in pregnancy following bariatric surgery [27]. We are not aware of any reports in the published literature of the use of other pharmaceutical agents for the treatment of post-prandial hypoglycemia, such as octreotide and diazoxide, in pregnancy.

Dumping syndromes may present a further challenge in pregnancy following bariatric surgery. An oral glucose tolerance test is recommended in many women who conceive following bariatric surgery as gestational diabetes risk may remain high due to ongoing obesity. However, this test may trigger severe early and/or late dumping syndromes, compromising maternal and fetal safety, and interpretation of results can be difficult. Our group has recommended capillary blood glucose monitoring daily before and after meals for a week at 24 to 28 weeks’ gestation as a safer alternative to the oral glucose tolerance test [66].

#### 3.2.7. Other Considerations

For pregnancy occurring following adjustable gastric banding, the band can be deflated to improve food tolerance, reduce vomiting/regurgitation and to avoid excessive weight loss in early pregnancy [52]. Due to gastrointestinal changes associated with pregnancy, some late postoperative complications may present for the first time in pregnancy. Delayed diagnosis may result from difficulty distinguishing these from pregnancy symptoms [52]. Notable surgical complications during pregnancy could include small bowel obstruction, internal hernias, gastric band erosion or migration and cholelithiasis [27].

Proton pump inhibitors are commonly prescribed to patients following bariatric surgery to treat symptoms of gastro-esophageal reflux, which can be exacerbated by pregnancy. In the UK, omeprazole is the favored proton pump inhibitor as it has the most data available to support its safety in pregnancy [67]. Non-steroidal anti-inflammatory drugs are commonly prescribed when analgesia is required following birth. However, these drugs may lead to an increased risk of gastric ulceration following bariatric surgery [68], and are best avoided.

## 4. Conclusions

Bariatric surgery is increasingly being used as a cost-effective method to treat obesity, providing the benefit of reduced risk of weight-related co-morbidities. In women of childbearing age there are additional benefits in terms of fertility and pregnancy outcomes. However, there is a higher risk of protein and calorie malnutrition and micronutrient deficiencies in pregnancy. It is advisable to delay pregnancy for at least 12 months, preferably 2 years, following bariatric surgery. Comprehensive pre-conception and antenatal care is essential to achieving the best outcomes. Nutrition in pregnancy following bariatric surgery requires specialist monitoring and management. A multidisciplinary approach to care is desirable with close monitoring for deficiencies undertaken at each trimester.

## Figures and Tables

**Table 1:** Summary of global recommendations for supplements post-bariatric surgery.

	Recommendation	Comments
Multivitamin and mineral supplement	1–2 daily	Avoid retinol-based vitamin A during pregnancy and lactation; safe to continue beta-carotene
Calcium	800–1500 mg daily	Calcium citrate may have better bioavailability
Vitamin D	800 units daily	Higher doses may be necessary if pre-existing deficiency
Iron	45–60 mg daily	100 mg elemental iron is recommended for menstruating women
Vitamin B12	1000 micrograms orally daily or 1000 micrograms intramuscular injection 4–12 weekly	
Thiamine (B1)	As contained in Multivitamin or 12–50 mg daily	Additional 200–300 mg if prolonged vomiting is experienced
Folic Acid	As contained in Multivitamin or 400–800 microgram daily	5 mg preconception to 12 weeks of gestation
Vitamin A	As contained in Multivitamin or 5000–1000 IU daily	Additional screening in BPD/DS * or if Steatorrhoea. Increased requirements in pregnancy—avoid retinol and retinyl esters.
Vitamin E	As contained in Multivitamin or 15 mg daily	Additional screening in BPD/DS * or if Steatorrhoea
Vitamin K	As contained in Multivitamin or 90–300 micrograms daily	Additional screening in BPD/DS * or if Steatorrhoea
Zinc	As contained in Multivitamin to meet 100–200% RDA ^†^	Maintain Ratio of 8–15 mg Zinc per 1 mg Copper
Copper	As contained in Multivitamin to meet 100–200% RDA ^†^	Maintain Ratio of 8–15 mg Zinc per 1 mg Copper
Selenium	As contained in Multivitamin	

Global recommendations based on published guidelines from America, the UK and Australia [30,31,32,34]. In Pregnancy, we recommend a daily oral complete multivitamin and micronutrient (avoiding retinol), calcium with vitamin D, iron and 3-monthly intramuscular Hydroxocobalamin; omeprazole is our preferred choice of proton pump inhibitor. * BPD/DS, biliopancreatic diversion/duodenal switch. ^†^ RDA, recommended dietary allowance.
